# Biphasic contrast-enhanced [^18^F]PSMA-1007 PET/CT imaging to improve the detection of local relapse of prostate cancer

**DOI:** 10.1186/s13550-025-01252-4

**Published:** 2025-05-30

**Authors:** Eduards Mamlins, Emil Novruzov, Tadashi Watabe, Yuriko Mori, Mardjan Dabir, Katalin Mattes-György, Christina Antke, Jan Henke, Matthias Boschheidgen, Julian Kirchner, Danny Jazmati, Jan Hausmann, Jan P. Radtke, Günter Niegisch, Jens Cardinale, Juliane Hörner-Rieber, Peter Albers, Gerald Antoch, Frederik L. Giesel, Lars Schimmöller

**Affiliations:** 1 Department of Nuclear Medicine, Medical Faculty and University Hospital Duesseldorf, Heinrich Heine University Duesseldorf, Moorenstrasse 5, 40225 Duesseldorf, Germany; 2 Institute for Radiation Sciences, Osaka University, Osaka, 565-0871 Japan; 3 Department of Diagnostic and Interventional Radiology, Medical Faculty and University Hospital Duesseldorf, Heinrich Heine University Duesseldorf, 40225 Duesseldorf, Germany; 4 Department of Urology, Medical Faculty and University Hospital Duesseldorf, Heinrich Heine University Duesseldorf, 40225 Duesseldorf, Germany; 5 Department of Radiation Oncology, Medical Faculty and University Hospital Duesseldorf, Heinrich-Heine-University Duesseldorf, 40225 Duesseldorf, Germany; 6 Department of Diagnostic, Interventional Radiology and Nuclear Medicine, Marien Hospital Herne, University Hospital of Ruhr University Bochum, 46625 Herne, Germany; 7 Centre for Integrated Oncology (CIO) Düsseldorf, CIO Aachen-Bonn-Cologne-Duesseldorf, 40225 Duesseldorf, Germany

**Keywords:** PSMA imaging, [^18^F]PSMA-1007, Late imaging, Biphasic PSMA imaging, Contrast enhancement, Local relapse

## Abstract

**Background:**

The implementation of PSMA imaging in prostate cancer (PC) management has significantly improved the medical care of patients owing to its clinical impact, particularly with respect to biochemical recurrence. However, there is still an unmet clinical need regarding the correct discrimination of equivocal, centrally located, focal [^18^F]PSMA-1007 uptake without any CT-morphological findings in the postsurgical prostate bed. The aim of this monocentric, retrospective study was to investigate the efficacy of a biphasic, contrast-enhanced [^18^F]PSMA-1007 acquisition protocol.

**Results:**

This study investigated a total of 24 biologically male patients with BCR, with a mean PSA level of 0.96 ng/ml at the time of recurrence. The presence of local relapse was regarded as consistent by biphasic, contrast-enhanced [^18^F]PSMA-1007 PET/CT scans, of which 22 cases were finally validated through the composite reference standard after a 2-years follow-up. The acquisition of whole-body, contrast-enhanced PET/CT imaging data was performed after a mean of 105 (± 19) minutes, whereas late-phase PET/CT imaging of the pelvis with low-dose CT was conducted after 140 min (± 10) on average following the intravenous application of [^18^F]PSMA-1007 (injected mean activity of 240 MBq (± 29)). The median SUV_max_ and SUV_mean_ values of local relapse increased by 26% and 5%, respectively, in late-phase images. Moreover, median TBR with respect to the obturator internus muscle seemed to benefit the most from late-phase imaging, with an increase of 185%. The dynamics of the SUV metrics and TBR in lesions were statistically significant (*P* value < 0.001–0.019). Moreover, the retrospective reading of delayed [^18^F]PSMA-1007 PET/CT imaging provided an upgrade of the reporting for suspected local PC relapse from a previous PSMA-RADS 3A to a later PSMA-RADS 5 in seven patients (29%), unless the impact of contrast agent in the urethra would also be considered equally important. For the remaining patients, the qualitative evaluation of contrast agent displacement in the urethra was necessary for a final clinical decision that provided the upgrading of the reporting to PSMA RADS 5 for an additional nine patients (38%).

**Conclusions:**

Given the aforementioned, highly specific unmet clinical need for a relatively small ratio of patients with prostate cancer undergoing PSMA imaging, our proposed acquisition protocol mandates a well-balanced preselection of patients. Under this premise, the study results demonstrated that the optimized acquisition protocol with biphasic contrast-enhanced [^18^F]PSMA-1007 PET/CT imaging improved the diagnostic performance for the detection of local PC recurrence in 67% of preselected patients.

**Supplementary Information:**

The online version contains supplementary material available at 10.1186/s13550-025-01252-4.

## Introduction

With the introduction of PSMA PET ligands a decade ago, the landscape of prostate cancer (PC) management has shifted profoundly, and accordingly, the international and national guidelines have appreciated the added value of PSMA imaging through its implementation in routine clinical practice [1–5]. The initially introduced ^68^Ga-labeled PSMA tracer has several limitations, such as limited batch production and suboptimal spatial resolution due to the wide positron range of ^68^Ga [6], which led to the development of various radiofluorinated PET tracers.

Among them, [^18^F]PSMA-1007 has proven to be an interesting tracer, with a distinct feature of up to 95% hepatobiliary excretion with 5% remaining renal excretion, offering favorable biodistribution and spatial resolution, especially for the detection of small lesions near the bladder and therefore local relapse [2, 7, 8]. By the beginning of 2024, [^18^F]PSMA-1007 had gained approval from regulatory authorities in eight western European countries following the successful completion of a clinical phase III study (ABX-CT-301) [9]. Therefore, its use is expected to increase by surpassing other PSMA PET ligands owing to its favorable pharmacokinetics compared with those of PSMA ligands with the renal elimination route. [^68^Ga]PSMA-11 represents the most employed PET tracer worldwide. Hence, most research efforts for acquisition protocol optimization have focused on this tracer. This approach included acquisition protocol amendments, such as the combination of forced diuresis with or without late imaging or the acquisition of postvoid late images without the application of forced diuresis. In particular, the combination of forced diuresis and late imaging has been demonstrated in several works to increase detection rates [10–13].

In addition, few studies have investigated the utility of the application of an intravenous contrast agent for better discrimination of the malignancy as a part of whole-body acquisition or special computed tomography (CT)-urography, which reveals positive results for suspected foci adjacent to ureters. In contrast, no additional benefit was recorded for lesions in the skeletal system or local relapse [14–18]. However, the adoption of these amendments to the standardized PET protocol regarding ^68^Ga-labeled PSMA ligands for [^18^F]PSMA-1007 is challenging because of their different pharmacokinetics, as the current literature indicates a significant scarcity of data regarding this issue.

Despite the advantage for the detection of local relapse in the postsurgical prostatic bed owing to a lack of spillover radioactivity from the urinary bladder, accurate discrimination of, in particular, a centrally located culprit PSMA uptake from focal PSMA ligand retention (radiourine) in the urethra within the prostate bed might be challenging in regular clinical care. The PSMA-RADS V1 reporting system depicts these equivocal lesions as PSMA-RADS 3A, necessitating further work-up to clarify the local findings [19–21].

To our knowledge, the only research group investigating dual-time PET acquisition via [^18^F]PSMA-1007, Rahbar et al. proposed a standard acquisition time of 120 min following the observation of a 70% increase in the median SUV_max_. The comparison of early and late acquisition times of 60 and 120 min, respectively, revealed that malignant lesions demonstrate continuous uptake of [^18^F]PSMA-1007 due to its highly lipophilic properties. These data only indirectly emphasize the potential of delayed images in [^18^F]PSMA-1007 [22]. Furthermore, the added value of contrast-enhanced [^18^F]PSMA-1007 PET/CT scans with potentially improved detection of local relapse in the prostate bed has been reported [18, 23].

The aim of this monocentric, retrospective study was to investigate the potential of a biphasic, contrast-enhanced [^18^F]PSMA-1007 acquisition protocol for discriminating equivocal, centrally located, focal [^18^F]PSMA-1007 uptake without any CT-morphological findings in the postsurgical prostate bed in selected patients.

## Materials and methods

### Patient population

A total of 24 biologically male patients who underwent dual-time contrast-enhanced [^18^F]PSMA-1007 PET/CT after biochemical recurrence (BCR) following curative-intent radical prostatectomy were enrolled in this monocentric, retrospective study between May 2021 and February 2022 out of 110 patients at a tertiary referral hospital. All patients underwent [^18^F]PSMA-1007 PET/CT scans with intravenous contrast enhancement and additional acquisition of delayed-phase scans due to equivocal focal [^18^F]PSMA-1007 uptake in the postsurgical prostate bed. The biochemical recurrence of PC was defined as an elevation in the prostate-specific antigen (PSA) level above 0.2 ng/ml in two consecutive follow-up controls or a persistent PSA value ≥ 0.2 ng/ml following definitive surgical therapy in accordance with current guidelines [3, 24]. The use of [^18^F]PSMA-1007 PET/CT scans with contrast enhancement is the standard institutional protocol in our clinic, whereas delayed scans of the pelvis are acquired only in patients with equivocal [^18^F]PSMA-1007 uptake in the prostate bed. Table 1 shows the patient characteristics.

**Table 1:** Baseline patient characteristics

Parameter	Value
Age (mean ± SD) in years	71 (± 5)
ISUP (Prior Treatment)	
ISUP 2	8
ISUP 3	10
ISUP 4	2
ISUP 5	4
Robot assisted Radical Prostatectomy	24
PSA nadir (median, range) in ng/ml	0.03 (0.01–0.06)
PSA at the time of PET/CT scan (median, range) in ng/ml	0.96 (0.21–16.5)
Biochemical recurrence free time (between surgery and PET/CT scan) (median, range) in months	50 (7–172)
Injected [^18^F]PSMA-1007 Activity (mean ± SD) in MBq	240 (± 29)
Acquisition time for early images (after IV tracer injection) (mean ± SD) in minutes	105 (± 19)
Acquisition time for late images (after IV tracer injection) (mean ± SD) in minutes	140 (± 10)

*ISUP* International society of urological pathology; *PSA* Prostate specific antigen

The data were pseudonymized and retrospectively analyzed. The study received approval from the Ethical Committee of the Medical Faculty of Heinrich Heine University Duesseldorf, Germany (Study-Nr.: 2022–1898).

### PET/CT acquisition

The acquisition of whole-body, contrast-enhanced PET/CT imaging data was performed after a mean of 105 (± 19) minutes, whereas late-phase PET/CT imaging of the pelvis with low-dose CT for anatomic localization and attenuation correction was conducted after 140 min (± 10) on average following the intravenous application of [^18^F]PSMA-1007 (injected mean activity of 240 MBq (± 29)). All PET/CT scans were acquired in 3D mode with a body weight-adjusted acquisition time of 3–5 min/bed position with a Siemens Biograph 128 mCT PET/CT scanner (Siemens, Erlangen, Germany) in accordance with our institutional protocol (Supplementary Table).

Image acquisition (early images) was performed in the supine position from the skull base to the mid-thigh or from the head to the feet as a whole-body PET/CT scan. The CT component was performed 70 s after intravenous injection of a weight-adapted dose of iodinated contrast agent with a maximal dose of 80 ml (Accupaque 300, GE Healthcare, Munich, Germany) followed by a 60 ml bolus of physiological saline. The subsequent PET scan was acquired in the caudocranial direction in accordance with national and international guidelines [4, 5]. A low-dose deep inspiration chest CT scan was also performed for better assessment of the lung tissue. The delayed PET/CT scan was acquired 140 (± 10) minutes on average after radiotracer injection for the pelvis region after urinary bladder emptying. No additional iodinated CT contrast agent was injected for late-phase PET/CT. All patients were monitored for any new symptoms or abnormalities up to 30 min after the end of the examination.

### Image analysis

Tracer uptake in lesions was quantified by the mean and maximum standardized uptake values (SUV_mean_ and SUV_max_). The tumor-to-background ratio (TBR) was derived by dividing the SUV_max_ of the tumor lesions by the SUV_mean_ of the skeletal muscle in the pelvis region (Obturator internus muscle) and the blood pool in the right common iliac artery. Spheric volumes of interest (VOIs) were placed over the suspected lesions and normal organs (muscle, vessel) on early- and delayed-phase PET/CT scans by an experienced nuclear medicine physician with 5 years of experience in PSMA PET/CT under the supervision of an attending board-certified nuclear medicine expert and an attending board-certified radiology expert via the dedicated reading software program Hermes Medical Imaging (Suite v6.1, Hermes Medical Solutions AB, Strandbergsgatan 16, 11251 Stockholm, Sweden).

Lesions that were visually suggestive of local relapse in the early images (PSMA-RADS 3A) were further investigated via assessment of tracer uptake by the culprit lesion and displacement of contrast agent within the urethra on the delayed images [19–21]. Stable or increasing tracer uptake or, alternatively, a better tumor-to-background ratio (TBR) in the postsurgical prostate bed accompanied by clear spatial delineation of the urethra owing to contrast agent displacement at the anatomical level of focal tracer uptake was regarded as the imaging confirmation of genuine tumor uptake in the prostate bed. Previous experience in molecular imaging regarded a variability of SUV measurements of up to 10% as normal [25]. Two board-certified nuclear medicine physicians independently read all the datasets and resolved any disagreements by consensus. Histopathology after salvage surgery is considered the gold standard, whereas clinical, biochemical, and radiological follow-up serve as composite reference standards.

### Statistical analysis

We used descriptive analyses for demographics, tumor characteristics, and tracer uptake. SUV metrics in tumor and normal tissues as well as TBRs were analyzed via paired t tests or Wilcoxon signed rank tests. A *P* value of < 0.05 was considered to indicate statistical significance. All the statistical analyses were performed via SigmaStat Version 3.5 (Systat Software, Inc., San Jose, CA, USA) and SigmaPlot Version 11.0 (Systat Software, Inc., San Jose, CA, USA) for graphical visualization.

## Results

### Patient demographics and imaging parameters

**Fig. 1:**
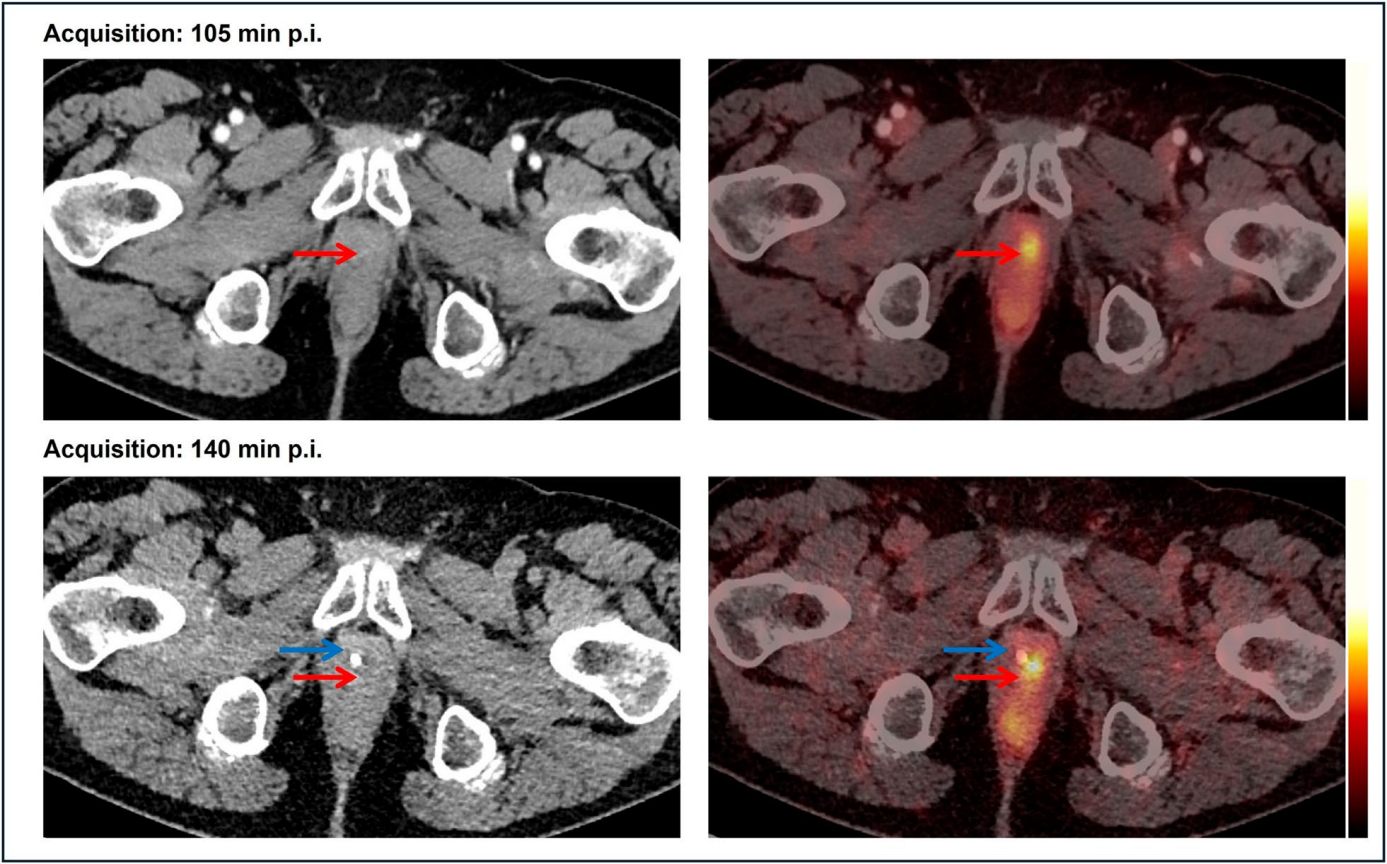
The juxtaposition of focal [^18^F]PSMA-1007 uptake to the urethra complicates the discrimination of culprit tracer uptake (red arrow) from residual radiourine in the urethra (blue arrow). In addition, the delayed image provided an increase in SUV metrics, leading to upgrade of the lesion from PSMA-RADS 3 A to PSMA-RADS 5

**Fig. 2:**
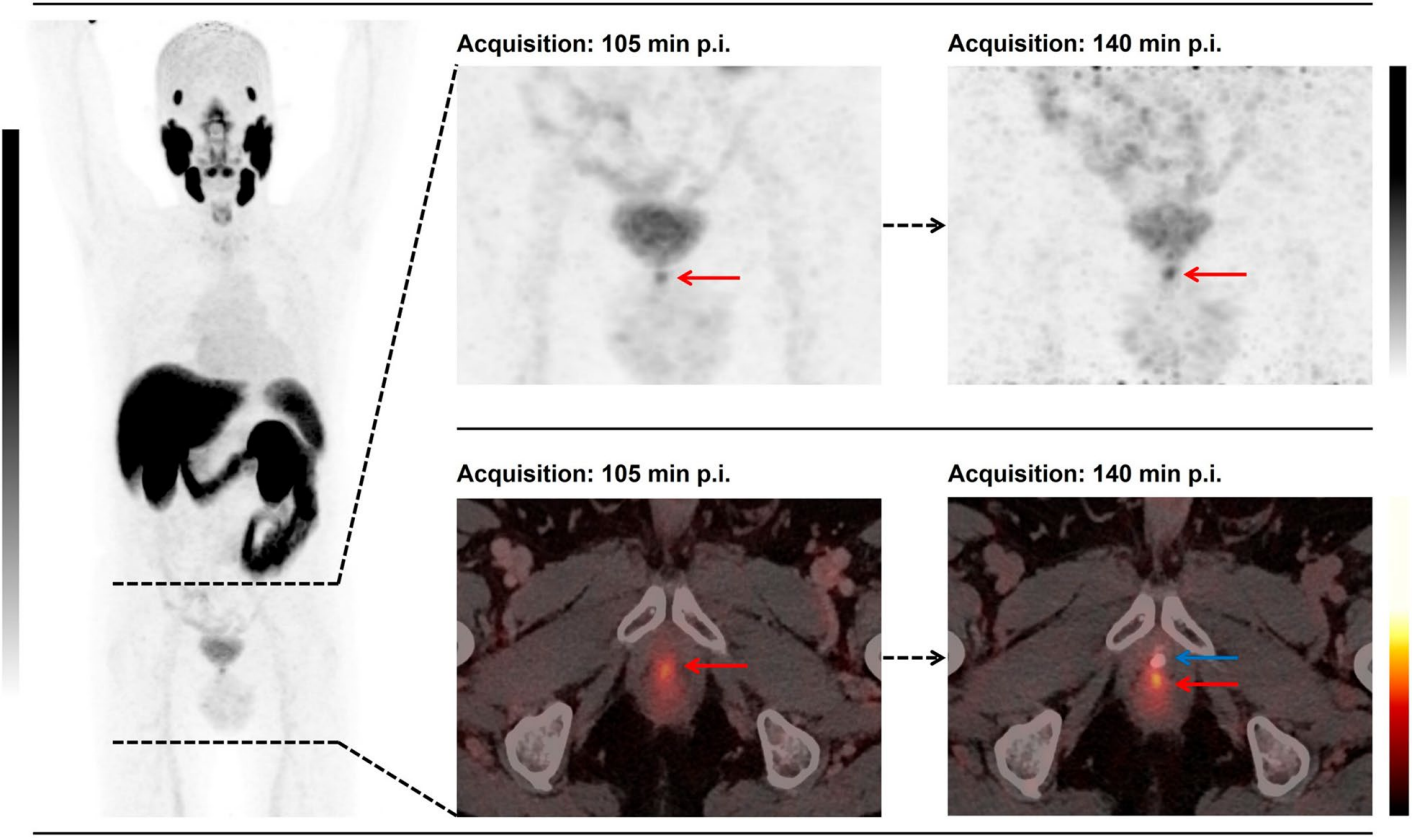
The juxtaposition of focal [^18^F]PSMA-1007 uptake (red arrow) to the urethra (blue arrow) complicates the accurate discrimination from residual radiourine in the urethra. With increasing tracer uptake of the lesion and detection of contrast agent in the urethra, an upgrading of PSMA-RADS, from 3 A to 5, could be reported

The study investigated a total of 24 biologically male patients in the setting of BCR, with a mean PSA level of 0.96 ng/ml at the time of recurrence work-up (Table 1). The presence of local relapse was regarded as consistent by biphasic, contrast-enhanced [^18^F]PSMA-1007 PET/CT scans, of which 22 cases was finally validated through the composite reference standard after a 2-years follow-up period. Suspected focal [^18^F]PSMA-1007 uptake was juxtaposed to the midline at the urethra, with a mean distance of 5 mm to the midline (range 0.1–25.0 mm) and a distance of 25 mm (range 0.1–65.0) cranially to the urogenital diaphragm. Figures 1 and 2 depict the clinically challenging discrimination of suspected focal PSMA uptake from the radiourine in the urethra.

**Fig. 3:**
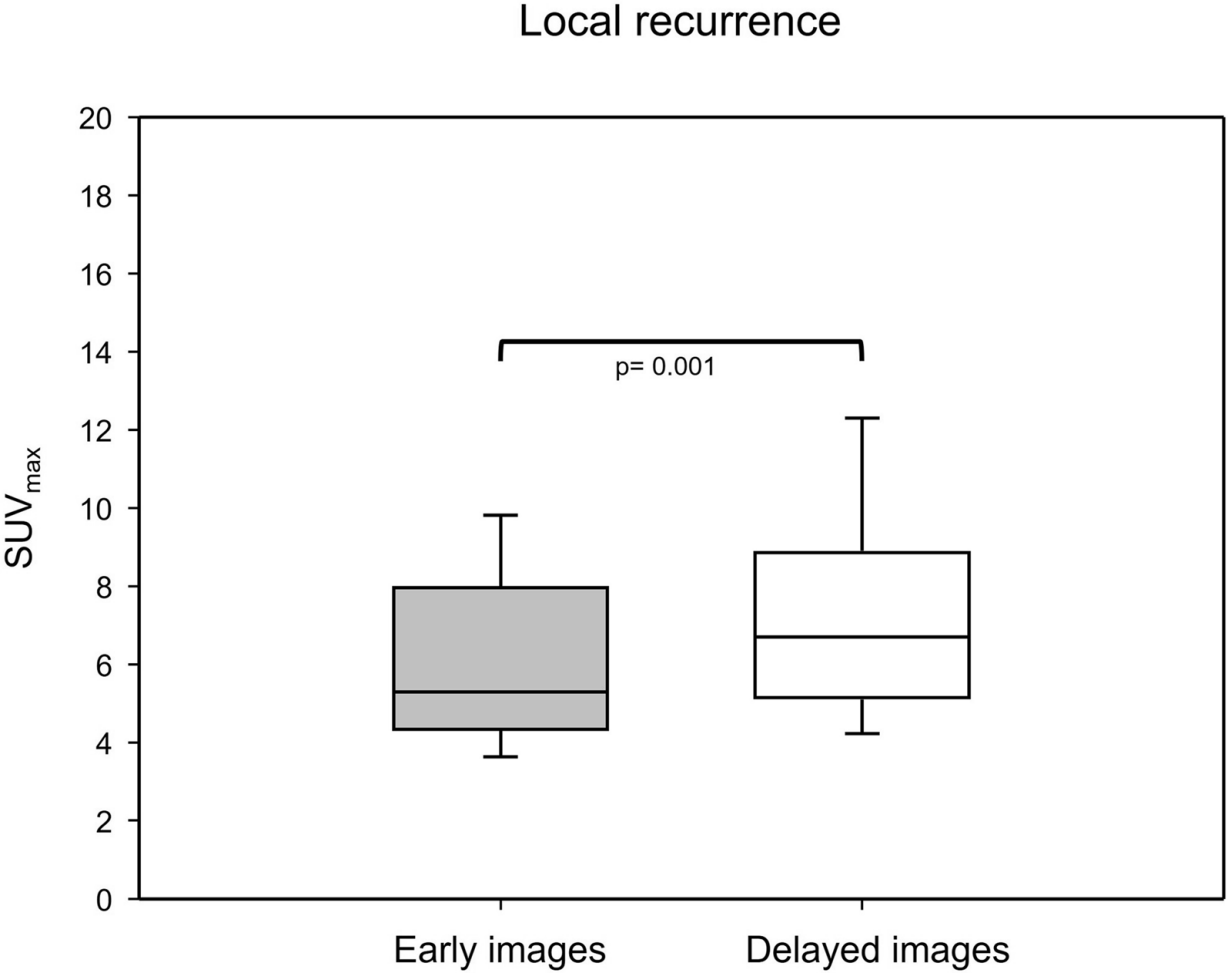
Comparison of the [^18^F]PSMA-1007 uptake of local recurrence in the early and delayed images that emphasized a statistically significant increase in the SUV_max_ in the delayed images (boxplots show IQRs; black lines show medians)

A standard early-phase, whole-body [^18^F]PSMA-1007 PET/CT scan was performed with contrast enhancement 105 (± 19) min after injection of [^18^F]PSMA-1007, with a mean activity of 240 (± 29) MBq. The retrospective reading of the early-phase images revealed an unequivocal detection of local relapse in only 7/24 patients (29%), with a median SUV_max_ of 5.3 (2.4–15.6). The median TBR with respect to the obturator internus muscle was 8.8 (4–31.2), whereas the median TBR with respect to the blood pool was 4.8 (2.2–10.4). We also measured tracer retention in the urinary bladder. The median SUV_mean_ was 5.6 (0.5–14.0) (Fig. 3).

### Impact of the acquisition of late-phase [^18^F]PSMA-1007 PET/CT imaging

**Fig. 4:**
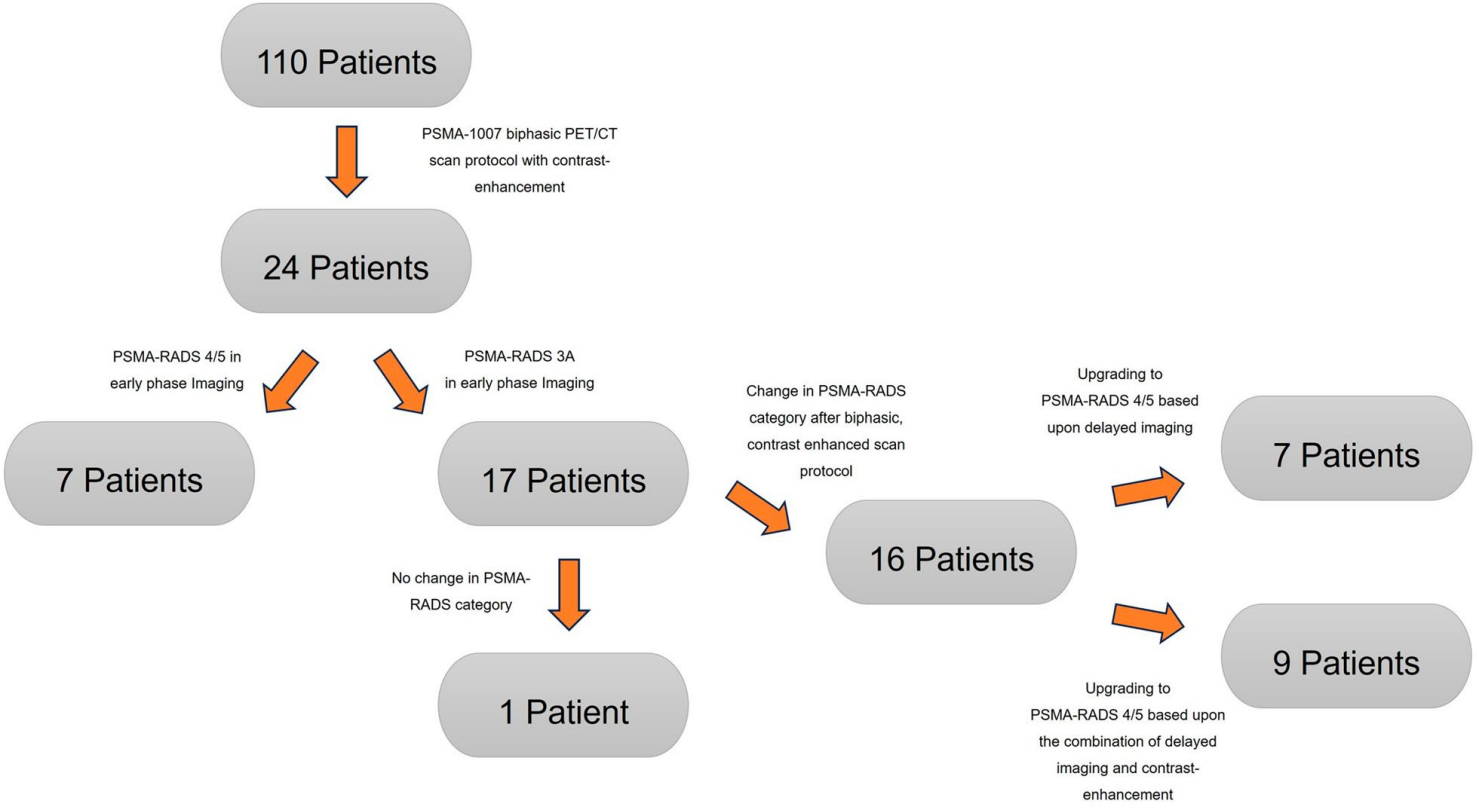
Flowchart depicts the patient enrollment and improvement of reporting by using the scan protocol of biphasic contrast-enhanced [^18^F]PSMA-1007 PET/CT

The acquisition of late-phase [^18^F]PSMA-1007 PET/CT imaging provided an upgrade of the reporting for suspected local relapse from a previous PSMA-RADS 3 A to a later PSMA-RADS 4/5 in seven patients (29%). Considering the additive impact of contrast agent in the urethra, this provided additional certainty in 9 patients (38%) in the delineation of local recurrence on late images (Fig. 4). In line with a previous study, we observed, however, a decrease in tracer uptake in the urinary bladder accompanied by an increase in the suspected lesion in terms of SUV metrics [22]. Accordingly, the SUV_max_ (median) and SUV_mean_ (median) of local relapse patients were increased by 26% and 5%, respectively, in late-phase images. Moreover, TBR (median) with respect to the obturator internus muscle seemed to benefit the most from late-phase imaging, with an increase of 185%. The dynamics of the SUV metrics and TBR in lesions were statistically significant (*P* value < 0.001–0.019).

These results seemed to be concordant with pharmacokinetic properties of [^18^F]PSMA-1007 [7]. Nevertheless, this cannot provide an enhancement of diagnostic performance for patients who exhibit a suspected lesion juxtaposed to the midline at the urethra (Fig. 1), as the tracer from the urinary bladder could have displaced to the urethra as well. Table 2 shows the dynamics of tracer uptake between standard early and delayed imaging. Since we acquired late images of only the pelvis region, a detailed, comparative analysis of biodistribution is beyond the scope of our investigation.

**Table 2:** Overview of the statistical analysis of the dynamics of SUV metrics and the TBR between early- and late-phase [^18^F]PSMA-1007 PET/CT images

	Early phase	Late phase	Dynamic (Δ) (%)	*P* value
*Local relapse*				
SUV_max_	5.3 (median)	6.7 (median)	+ 15	0.001*
SUV_mean_	4.5 (mean)	5.0 (mean)	+ 11	0.019*
TBR skeletal muscle	11.65 (median)	17.58 (median)	+ 50	< 0.001*
TBR blood pool	4.8 (median)	6.4 (median)	+ 33	< 0.001*
*Urinary bladder*				
SUV_max_	7.4 (median)	6.4 (median)	− 13.5	0.343
SUV_mean_	5.6 (median)	4.3 (median)	− 23.2	0.288

*TBR* Tumor-to-background ratio

*Statistically significant

### Added value of contrast enhancement in late-phase [^18^F]PSMA-1007 PET/CT imaging

As mentioned above, the assessment of the SUV metrics of late-phase imaging with the standard reading system improved the PSMA-RADS category in seven (29%) patients. For nine patients (38%), the qualitative evaluation of contrast agent displacement in the urethra was necessary for a final clinical decision that provided the upgrading of the reporting to PSMA RADS 5 (Table 3). In one patient, the focal lesion in the prostate bed remained unclear (Fig. 4).

**Table 3:** Overview of the impact of individual components of [^18^F]PSMA-1007 PET/CT acquisition on the upgrading of patients according to the PSMA-RADS reporting system

Impact of PET/CT acquisition	PSMA RADS category	Number of patients	Upgrade in %
Late phase	PSMA-RADS 5	7	29
Late phase + contrast enhancement in urethra	PSMA-RADS 5	9	38
Upgraded to PSMA-RADS 5		16 (out of 24 patients)	67

Owing to the caudocranial acquisition of [^18^F]PSMA-1007 PET/CT, there is a limited time window for the accumulation of contrast medium in the urinary bladder, which would subsequently displace into the urethra. Moreover, similar to other molecular imaging modalities, a CT morphological finding or correlation for suspected focal PSMA uptake is very unlikely, as PSMA imaging has been performed in BCR patients with very low PSA levels. Thus, we exploited the secondary effect of contrast medium within delayed imaging and thereby enhanced the interpretive value of this approach in combination with delayed-phase images for an overall improvement of 67%.

## Discussion

PSMA imaging, particularly [^18^F]PSMA-1007 PET/CT imaging, has been proven to be a very valuable molecular imaging modality with an excellent overall detection rate of up to 94% in the setting of BCR, outperforming conventional CT and/or MRI imaging and bone scanning for relapse [26]. In particular, for the detection of local recurrence, [^18^F]PSMA-1007 imaging has been reported to have a sensitivity and specificity of 94% and 92%, respectively [7, 27]. Local recurrence is termed for any lesion detected in the prostate bed, which is, in turn, a broader and, to some extent, vague definition encompassing anatomically a large area. Despite ongoing discussions regarding the boundaries of the postsurgical prostate bed, the current consensus is that the contours of the prostate bed extend cranially from the vesicourethral anastomosis (VUA) to the seminal vesicles bed in the anterior direction and to the posterior margin of the bladder wall in the posterior direction, as well as between the posterior margin of the pubic bone and the anterior rectum wall in the caudal direction. Additionally, the prostate bed is situated between the medial margins of the internal obturator muscles in the lateral direction [28]. Although [^18^F]PSMA-1007 is predominantly excreted over the hepatobiliary tract, it is, to some extent, also excreted through the renal pathway, showing accumulation in the urinary tract, including the urethra [8].

In our clinical experience, we detected an unmet clinical need for the accurate discrimination of equivocal, focal [^18^F]PSMA-1007 uptake juxtaposed to the urethra; as such, [^18^F]PSMA-1007 foci without any CT morphological findings pose challenges in regular clinical care because of the lack of clear delineation from the radiourine within the urethra. Gelikman et al. pointed out a correlation between anatomical features of urethra, e.g. larger dimension and higher incidence of severe urethral curvature, and midline PSMA radiourine stagnation in prostate bed [29]. This monocentric, retrospective study with a cohort of 24 patients presents an acquisition protocol with a combination of biphasic [^18^F]PSMA-1007 PET/CT, early and delayed images, and contrast enhancement by analyzing the added value of its individual components as well as the overall impact on diagnostic performance.

Since the implementation of PSMA imaging, several research groups have conducted clinical trials to optimize acquisition protocols, as most PSMA PET ligands are excreted via the renal pathway, potentially interfering with accurate discrimination of suspected findings in the pelvis and prostate bed. To overcome this obstacle, a number of strategies have been proposed, such as the acquisition of late images or the combination of standard acquisition with forced diuresis or contrast enhancement via CT-urography. These trials have been conducted with Ga-labeled PSMA-PET ligands with varying success rates. Beheshti et al. reported in a prospective, monocentric study with 50 patients an increasing tracer uptake of suspicious lesions for lymph node metastases and local relapse but a decreasing tracer uptake for bone metastases in late-phase [^68^Ga]PSMA-11 scans, albeit without any added value for diagnostic performance [30]. Schmuck et al. demonstrated in a retrospective, monocentric study with 240 patients an increasing target-to background ratio on delayed images of [^68^Ga]PSMA I&T. However, this had no significant effect on the detection rate [31]. The study by Hoffmann et al. supports the results of Beheshti et al. by underscoring the lack of added effects of late-phase images in the clinical workup [17].

Nevertheless, the aforementioned studies analyzed the effect of the use of undifferentiated late images for all the enrolled patients; thus, this fact might have compromised the diagnostic power of those results. In contrast, Afshar-Oromieh et al. designed a study protocol of a biphasic [^68^Ga]PSMA-11 scan with the use of diuretics and hydration in a retrospective study including 112 patients, where the clinicians performed the late-phase scan only for patients with equivocal findings. The group demonstrated a substantial improvement in the discrimination of indifferent findings after targeted patient selection for further late-phase scans [32]. This report was supported by the results of the research group of Morawitz et al., as this underscored the better lesion delineation due to a more favorable TBR [13]. The observation of mixed tracer uptake in various metastases of PC has been shared by the research groups of Afshar-Oromieh et al. and Alberts et al., who noted that not all lesions with a uniformly increasing tracer uptake pattern should be regarded as definitive criteria for malignancy. This might be a pitfall, especially for bone lesions [11, 32]. Although this pattern was observed with Ga-labeled PSMA ligands, we also experience a similar tracer uptake pattern for [^18^F]PSMA-1007 in our clinical practice. This needs further investigation.

Our institution protocol foresees the application of concurrent contrast-enhanced diagnostic CT for standard early [^18^F]PSMA-1007 PET/CT scans. There are few data in the literature concerning the deployment of concurrent contrast-enhanced diagnostic CT for PSMA PET/CT, which is somewhat intriguing. The integration of iodinated contrast medium in the PET/CT scan protocol, such as CT-urography (CTU), has been suggested by the research groups of Will et al. and Rosar et al., with promising results in preselected cases due to better delineation of suspected lesions in the vicinity of the ureters in [^68^Ga]PSMA-11. This involves an additional diagnostic scan 10 min after the IV administration of contrast agent following the standard, unenhanced PET/CT scan with low-dose CT [14, 15]. Despite the time-consuming patient preparation and the need for meticulous execution of the acquisition protocol, this might be a promising procedure for certain cases with indifferent findings juxtaposed to ureters for PSMA PET ligands with renal clearance. Trinh et al. compared the potential of concurrent contrast enhancement as an integral part of the standard early imaging of [^18^F]DCFPyL PET/CT scans with that of unenhanced, low-dose [^18^F]DCFPyL PET/CT scans. This study suggested a probable added value for subcentimetric lesions in the initial stages, whereas the effect of contrast enhancement in advanced stages revealed no benefit [33].

The only study investigating the application of contrast enhancement in [^18^F]PSMA-1007 imaging was conducted, to the best of our knowledge, by Tulipan et al., who discovered upward displacement of the tracer in the bladder due to contrast agent, improving visualization of the areas of the prostate bed adjacent to the posterior wall of the urinary bladder [23]. They realized this effect as a secondary effect due to the craniocaudal acquisition direction, which allows the accumulation of contrast agent in the urinary bladder and the generation of sediment on the posterior bladder wall with subsequent displacement of activity. The authors suggest that reversing the acquisition direction would increase the diagnostic certainty for some lesions in the prostate bed. However, this protocol has the disadvantage of an anatomical mismatch between PET and CT because of the bladder filling during examination, as the current guidelines recommend a caudocranial acquisition direction [4, 5].

In this study, we investigated the efficacy of a refined, contrast-enhanced [^18^F]PSMA-1007 PET/CT scan with a subsequent unenhanced delayed image of the pelvis for a dedicated diagnostic of the prostate bed. In light of the aforementioned literature data, we might expect a positive added value of biphasic imaging for the detection of local relapse in the prostate bed. In a cohort of 24 patients, equivocal tracer uptake adjacent to the urethra was detected in 17 patients. With the abovementioned contrast-enhanced [^18^F]PSMA-1007 PET/CT acquisition protocol including delayed images, we detected an overall increase in the PSMA-RADS category in 16 (67%) of the patients (Table 3). The combination of favorable physical features of ^18^F and pharmacokinetics of [^18^F]PSMA-1007 owing to its high lipophilicity appear to enhance its diagnostic performance [7, 8].

Delayed images alone contributed to the upgrading of the PSMA-RADS category in 7 patients (29%). The other cases with focal tracer uptake with poor delineation from the urethra have been challenging to discriminate, which was later only possible on the basis of the anatomical correlation of the contrast agent within the urethra and focal tracer uptake. This enabled an additional upgrade rate of 38% (9 patients). Notably, we did not exploit the primary function of the contrast medium to detect indifferent foci, but the secondary effect of displacement into the urethra has been exploited. In light of the early diagnosis of very low levels of PSA, we observed mostly no CT morphological findings for local relapse. Thus, the primary gain of contrast enhancement in standard early imaging is restricted to better delineation of pelvic lymph nodes in primary as well as restaging. This refined acquisition protocol had a positive outcome, with 67% of PSMA-RADS categories upgraded, which spared further costly and time-consuming follow-up examinations for our patients. The potential downsides of this protocol include potential conflicts with reimbursement by medical care providers owing to increased examination and personnel costs and potential contrast agent-related risk factors, such as allergic reactions, hyperthyroidism (if thyroid disorders are present) and kidney insufficiency. Another, to our opinion rather positive, side effect of this protocol is the enhancement of cooperation between nuclear medicine and radiology experts owing to the need for complex reading scheduling, as this is expected to increase the quality of hybrid imaging in regular clinical care.

The limitation of our study was its retrospective design with a relatively small patient cohort. Additionally, we cannot conduct a thorough comparative analysis of our results with those of other studies because similar studies for [^18^F]PSMA-1007 imaging are lacking. Moreover, all the cases were strongly preselected, with an inevitable selection bias for our cohort. However, to achieve optimal outcomes in the era of individualized medicine, we are obliged to perform adequate patient preselection. Otherwise, our protocol would most likely not work as efficiently as in this work, in case this would be deployed routinely for every patient with BCR. A per-patient follow-up time of at least 2 years is a substantial strength of our study. Furthermore, we plan to launch a mono-centric, prospective study in our center (NCT06657131; registration date: 23 October 2024) to analyze the efficacy of [^18^F]PSMA-1007 PET/CT imaging for BCR in a real-world setting with a larger patient cohort.

## Conclusions

This study demonstrates the value of a biphasic, contrast-enhanced [^18^F]PSMA-1007 PET/CT acquisition protocol for the accurate discrimination of equivocal tracer uptake in the prostate bed. The findings highlight the clinical utility of this patient-tailored approach, particularly in preselected cases of biochemical recurrence with the diagnostic challenges of focal [^18^F]PSMA-1007 uptake juxtaposed to the urethra, enabling a clearer distinction between malignant lesions and radiotracer accumulation.

## Supplementary Information

Additional file 1.

## Data Availability

The data used and/or analyzed during the current study are available from the corresponding author upon reasonable request.
