# Supplementary material for: Biphasic contrast-enhanced [18F]PSMA-1007 PET/CT imaging to improve the detection of local relapse of prostate cancer
Source: EJNMMI Res. 2025 May 30;15:61. doi: 10.1186/s13550-025-01252-4 (PMC12125407; doi:10.1186/s13550-025-01252-4)
Supplement: Supplementary file 1 — Additional file 1. [file 13550_2025_1252_MOESM1_ESM.docx]

**Supplementary Table**

| Modality | Low dose CT | Full dose (ce)CT |
| --- | --- | --- |
| CT reference (mAs) | 40 | 190 |
| CT peak kilovoltage (kV) | 120 | 120 |
| CT slice thickness (mm) | 1,5 | 1,5 |
| CT slice increment (mm) | 1 | 1 |
| PET reconstruction | OSEM algorithm | OSEM algorithm |
| Iterations | 4 | 4 |
| Subsets | 8 | 8 |
| Matrix | 200 x 200 | 200 x 200 |
| Corrections | Gaussian FWHM 2.0 mm | Gaussian FWHM 2.0 mm |
